# Sonochemical Synthesis of Sulfur Doped Reduced Graphene Oxide Supported CuS Nanoparticles for the Non-Enzymatic Glucose Sensor Applications

**DOI:** 10.1038/s41598-017-02479-5

**Published:** 2017-05-30

**Authors:** Natarajan Karikalan, Raj Karthik, Shen-Ming Chen, Chelladurai Karuppiah, Arumugam Elangovan

**Affiliations:** 1 Electroanalysis and Bioelectrochemistry Lab, Department of Chemical Engineering and Biotechnology, National Taipei University of Technology, No. 1, Section 3, Chung-Hsiao East Road, Taipei, 106 Taiwan (ROC); 2 Department of Chemistry, National Taiwan University, No. 1, Section 4, Roosevelt Road, Taipei, 106 Taiwan (ROC); 3 Department of Chemistry, Thiagarajar college, Madurai, Tamilnadu, 625009 India

## Abstract

Over the present material synthesis routes, the sonochemical route is highly efficient and comfortable way to produce nanostructured materials. In this way, the copper sulfide (CuS-covellite) and sulfur doped reduced graphene oxide (S-rGO) nanocomposite was prepared by sonochemical method. Interestingly, the structure of the as-prepared S-rGO/CuS was changed from the covellite to digenite phase. Herein, the S-rGO was act as a mild oxidizer and liable for the structural transformations. These structural changes are sequentially studied by various physicochemical characterizations such as Fourier transform infrared spectroscopy (FT-IR), Raman spectroscopy, X-ray diffraction (XRD), X-ray photoelectron spectroscopy (XPS) and Transmission electron microscopy (TEM). After scrupulous structural evaluations, the transformation of CuS phase was identified and documented. This oxidized CuS has an excellent electrocatalytic activity when compare to the bulk CuS. This S-rGO/CuS was further used for the determination of glucose and acquired good electrocatalytic performances. This S-rGO/CuS was exhibited a wide linear concentration range, 0.0001–3.88 mM and 3.88–20.17 mM, and a low-level detection limit of 32 nM. Moreover, we have validated the practicability of our developed glucose sensor in real biological samples.

## Introduction

Sonochemistry is an efficient route to synthesize the nanomaterials with the bulk production of new or existing materials, which provides a unique chemical route to furnish nanomaterials without high pressures, high temperatures and prolong reaction conditions^1^. This sonochemical reaction works on different kinds of phenomena especially the acoustic cavitation (the formation, growth, and collapse of bubbles)^2^. Actually, it is employed in the synthesis of nanomaterials or modification of solids and polymers/biopolymers. So far, enormous nanomaterials have prepared by sonochemistry albeit the synthesis of hetero atom doped graphene materials are highly demanded^3^. Thus, it is necessary to account the synthesis of hetero atom doped graphene by sonochemical route. Herein, the sulfur doped graphene was chosen due to their remarkable physicochemical properties and that makes it an efficient catalyst for the hydrogen storage, ORR, supercapacitors and batteries^4^. Besides, it has the mild oxidizing power which oxidizes the metal chalcogenides (CuS) in aqueous solutions.

**Figure 1:**
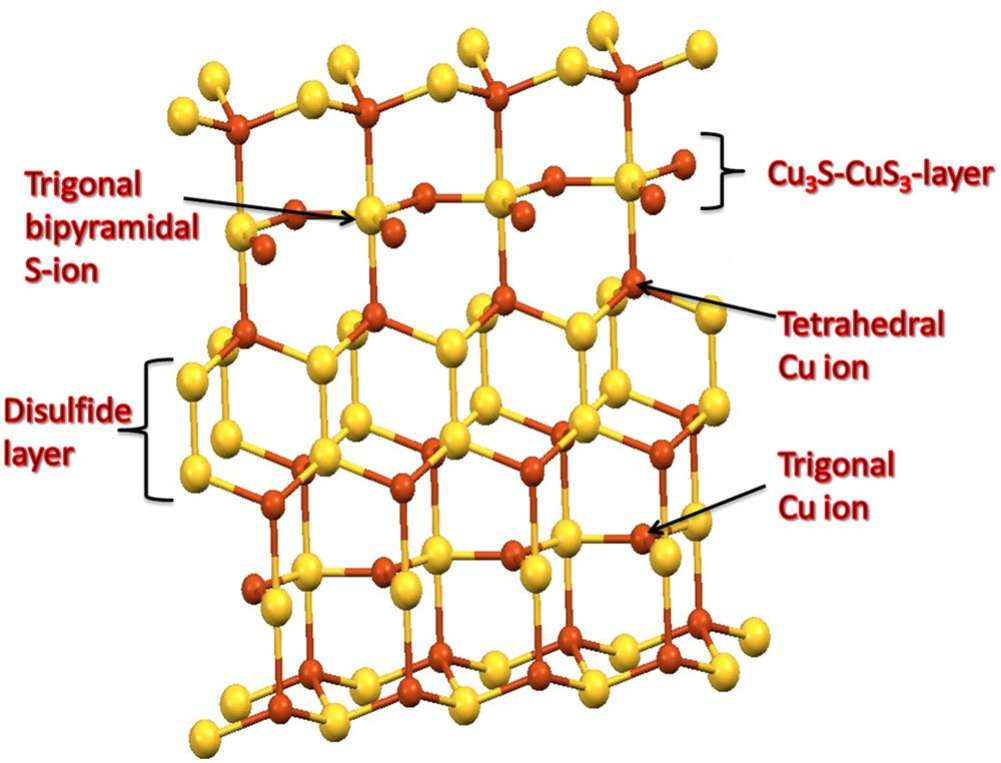
The crystal model of copper sulfide (covellite).

The CuS (covellite) is known as an extensively characterized inorganic compound and has a complex structure^5^. Commonly, the stoichiometric ratio of the CuS was forced to think the valance state of the Cu is (Cu^2+^ S^2−^). But, the CuS has been described as the mixed valance states of copper with the disulfide bridge (Cu^2+^ S_2_^2-^) (Cu_2_^+^ S^2−^)^6^. Actually, the later research studies were confirmed that the CuS consists of only Cu (I) by L-edge XAS^7^, XPS^8^ and theoretical studies^9^. In accordance, the structure of the CuS was proposed as (Cu_3_^+^ S_2_^2−^ S^2−^), however the charge of the compound is not balanced, hence the positive “hole” was created throughout the lattice. This is the reason for the “p type” semiconducting behavior of CuS^10^. Thus, the Cu (II) precursor was reduced in aqueous solutions when formed as a sulfide. The complete structure of the CuS was shown in Fig. 1, where two Cu ions are in trigonal coordination and four Cu ions are in tetrahedral coordination. The single S ions are surrounded by the Cu ions in trigonal bi-pyramidal coordination and the remaining S ions are forming a disulfide (S-S) groups, which bridging the two Cu_3_S-CuS_3_ layers^11^. In this study, we have investigated the structural changes of CuS by using the sulfur doped reduced graphene oxide (S-rGO). Followed by our previous work, we scrutinized the oxidizing properties of S-doped rGO that influences the oxidizing behavior from loan pairs of sulfur^12^. The structural changes were confirmed by various physicochemical characterizations and the electrochemistry of that compounds were examined towards the glucose oxidation.

The glucose is considered as a vital compound for the cellular metabolism but uncontrollable glucose level causes diabetes mellitus^13^. Therefore, the detection of glucose level in the blood is essential for the regular physiological fitness. A numerous analytical techniques have been developed to determine the glucose such as optical, acoustic, electronic, transdermal, fluorescent and electrochemical methods^14–19^. Among all, the electrochemical method provides rapid and cost effective platform to the determination of glucose. There are two electrochemical routes such as enzymatic and non-enzymatic determinations are possible to determine the glucose. However, the enzymatic glucose sensors have some drawbacks from the temperature, pH, oxygen and humidity^20^. Hence, the non-enzymatic glucose determination was developed for alternative to enzymatic glucose sensors. There are many materials have been reported as glucose sensor electrode which includes Co, Cu, Ni, Co_3_O_4_, NiO, WO_3_, CdO/CuO, TiO_2_, Mn_3_O_4_, MnO_2_, Fe_2_O_3_ and ZnO, etc^21^. However, these sensor electrodes has to be still improved from its sensitivity, selectivity and stability. Hence, we have rectified those issues and developed the efficient sensor electrode based on the S-rGO/CuS composite and the obtained analytical performances were comparable with previous work.

## Results and Discussion

### Structural characterizations

**Figure 2:**
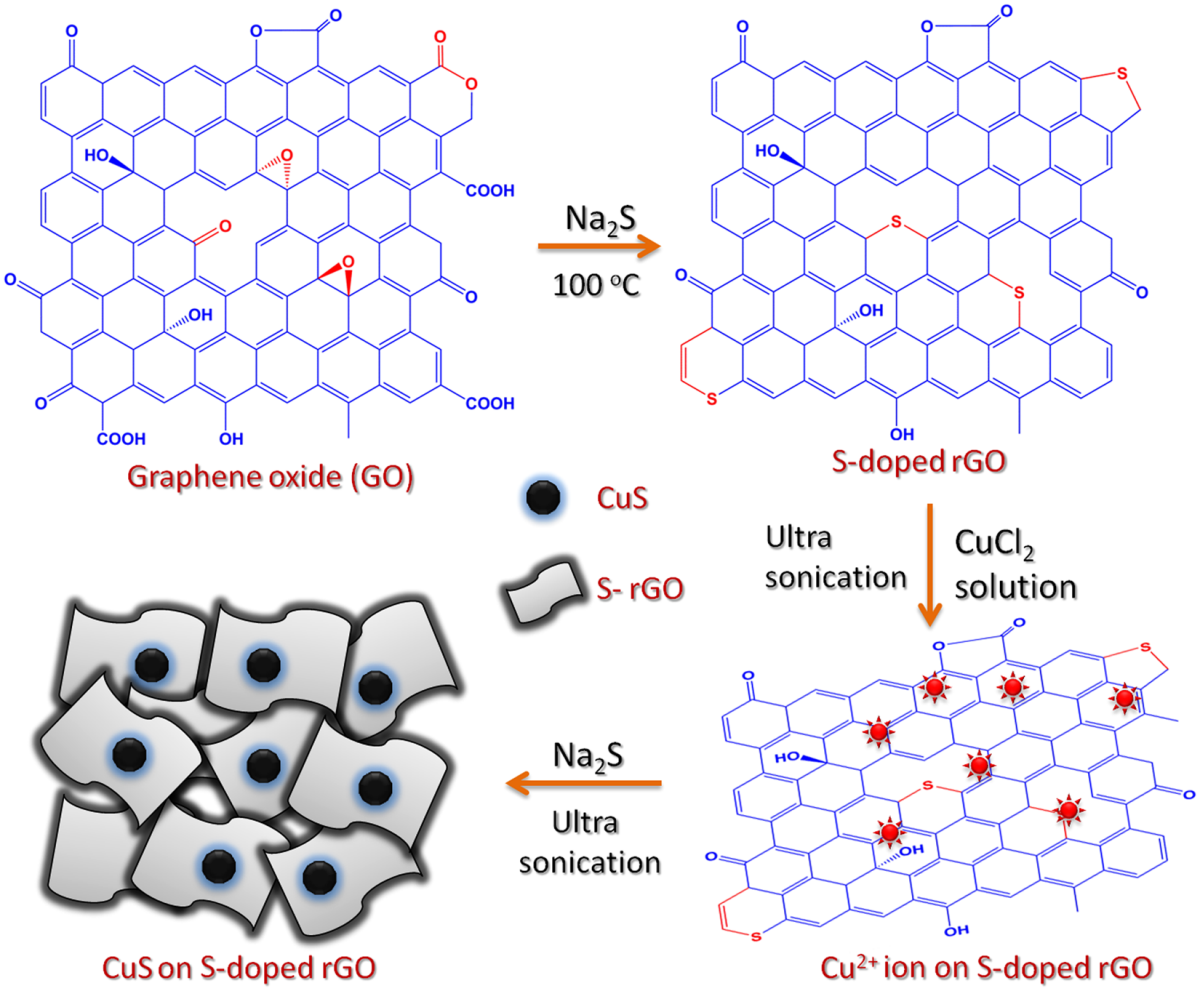
The schematic view of the overall sonochemical synthesis procedure for the preparation of S-rGO/CuS.

The sonochemical synthesis of S-rGO was simpler, however the mechanism of the S-rGO formation was quite complex. The Raman and XPS analysis are the most sophisticated tool to examine the carbonaceous materials. Hence, the formation of S-rGO was primarily examined by Raman spectra and later the S dopant was confirmed by XPS. The Raman spectrum of S-rGO (Fig. S1) was revealed two broad bands at 1365 and 1596 cm^−1^, for the D band (disordered carbon) and G band (graphitic carbon). Generally, the D band corresponds to the breaking of sp^2^ symmetry and the G band corresponds to the first-order scattering of the stretching vibration mode (E_2g_) of sp^2^ carbon domains^22^. The D band intensity of the S-rGO was higher than that of G band which reveals that there is a structural collapse in sp^2^ carbon lattice. This structural collapse was created by the reduction of oxygen functionalities in GO, further, the insertion of sulfur ion leads to some disorders. The relative intensity ratio for the D and G bands (I_D_/I_G_) of S-rGO is 1.15 whereas the GO reveals ~1^23^. This result indicated that the oxygen functionalities of GO was partially reduced and the sulfur ion was inserted into the carbon lattices^24^. The chemical environment and the S dopant was further confirmed by XPS analysis. Figure S2 shows the high-resolution C 1s spectrum of S-rGO, which further deconvoluted into four peaks. The signals at 284.9, 286.4 and 289.3 eV are attributed to the C–C, C=O and O–C=O bonding environments which are the typical characteristics commonly present in carbonaceous compounds^25^. The signal at 285.7 eV corresponds to the C–O or C–S, which covers the maximum area under the curve, hence it evinced that the sulfur ion was doped in carbon lattice^23, 25^. Furthermore, the high-resolution S 2p spectrum was assessed to confirm the S-dopant in S-rGO. Figure S3 shows high-resolution S 2p spectrum where the peaks at 161.89, 162.98, 164.17 and 168.67 eV are corresponds to C–S–C, C=S, C–S=O and sulfate bonding, respectively^26^. From that results, we confirmed that the sulfur ion was successfully doped in carbon lattice. Finally, the FT-IR spectra were used to evaluate the functional groups in S-rGO. Figure 4B shows the FT-IR spectrum of S-rGO (b), where the peaks at 3471, 1630, 1591 and 1408 cm^−1^ are attributed to the stretching vibrations of OH^−^, C=O, C=C and O=C–C, respectively^27, 28^. Further, the peaks at 1195, 900 and 580 cm^−1^ are corresponds to the vibrations of C–S–C, C–S and C–C=S^27, 28^. This result was supported to the formation of S-rGO accompanied with XPS analysis. From the above results, we attempt to propose a mechanism for the formation of S-rGO. Conversely, the signal for C–O–C bonding was absent in S-rGO, which stated that the epoxide group of GO might be converted into C–S–C or C–S. Moreover, the carbonyl group was highly electrophilic nature than that of other oxygen functionalities. Hence, the S^2−^ ions (from Na_2_S) attack the epoxide group or carbonyl group by the neculeophilic substitution which is the key step in the formation of S-rGO (the possible reactive groups and products are marked as red in Fig. 2).

**Figure 3:**
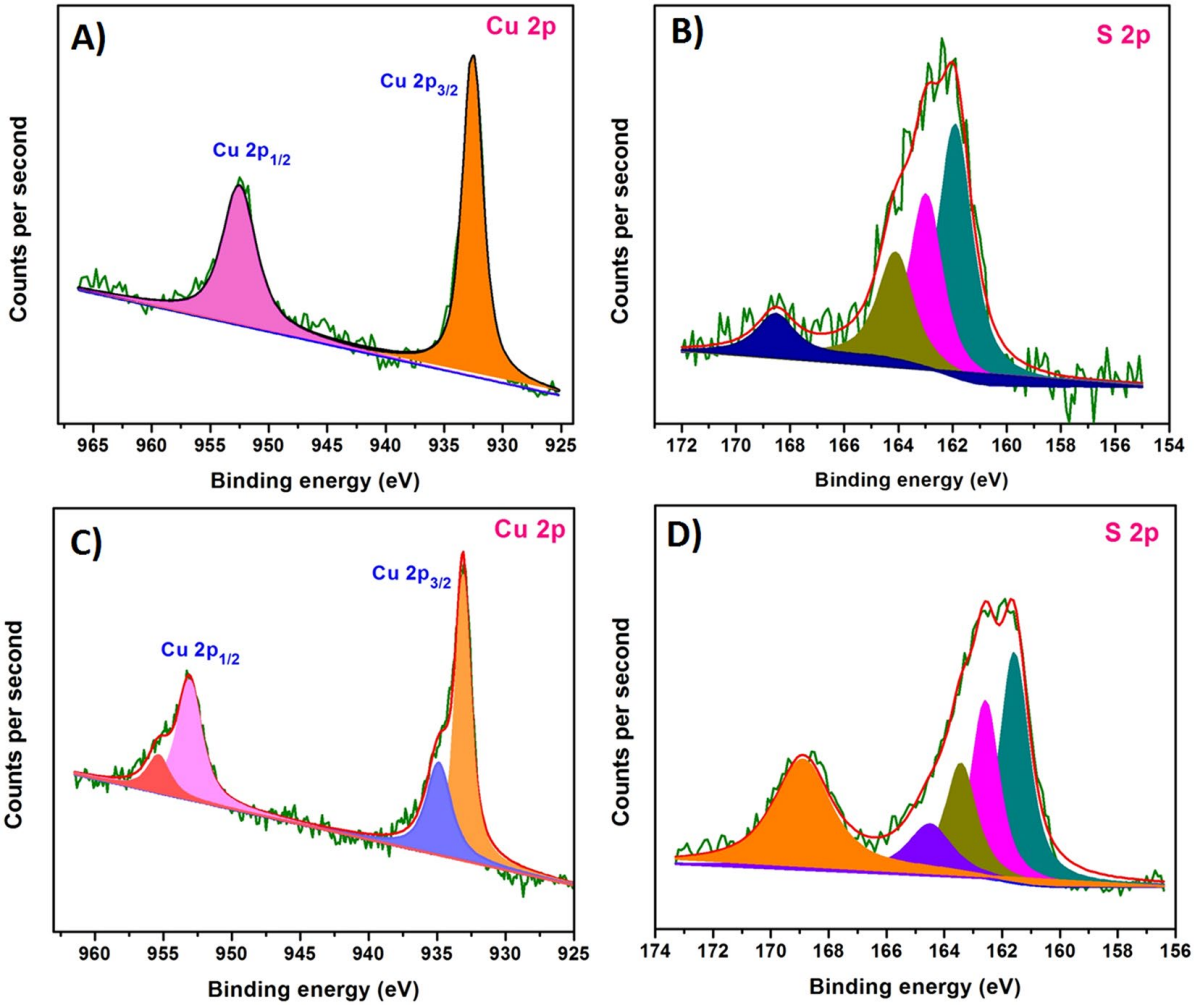
High resolution XPS spectrum of Cu 2p (**A**), S 2p (**B**) for the bulk CuS and Cu 2p (**C**), S 2p (**D**) for the S-rGO/CuS.

**Figure 4:**
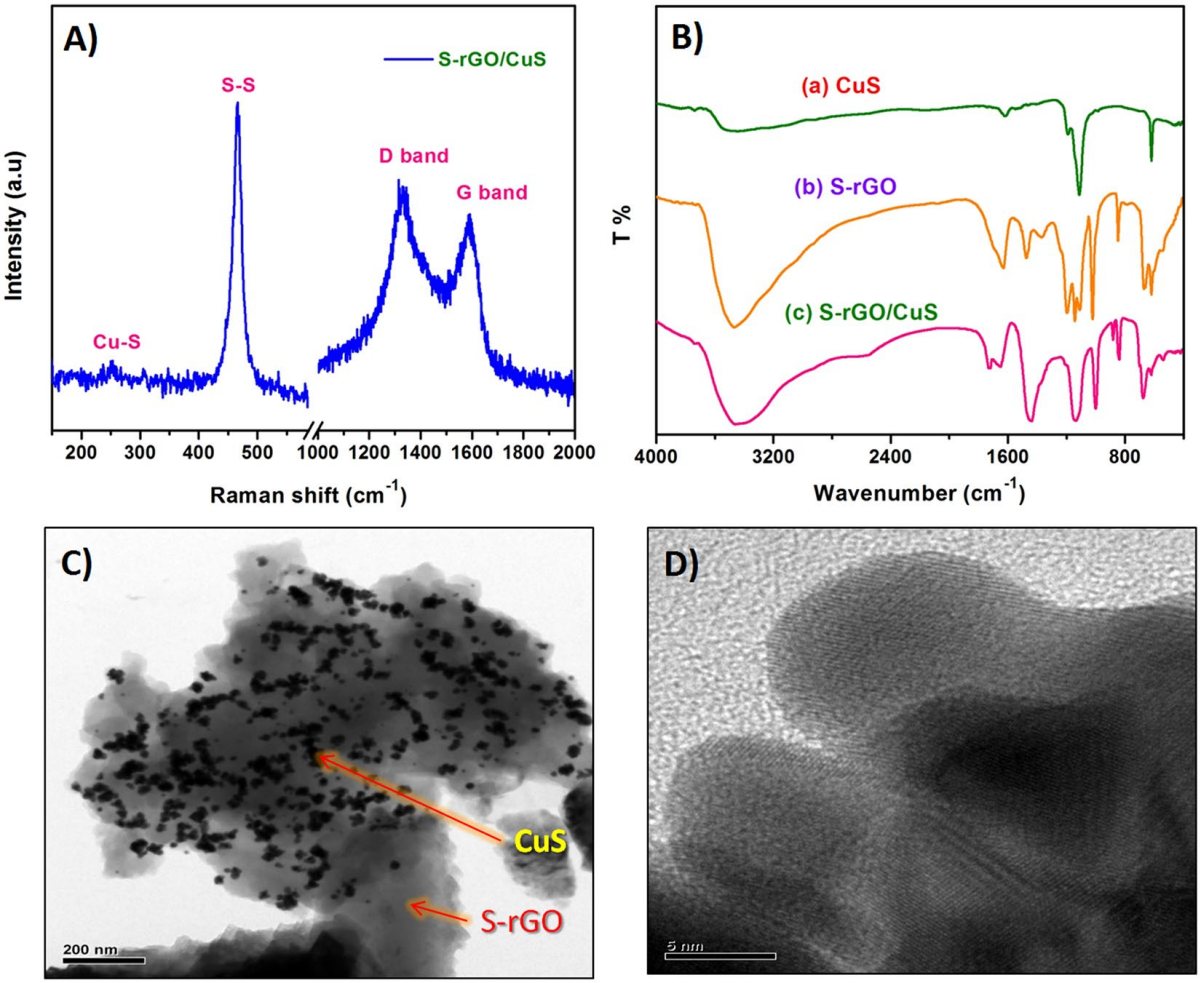
Raman spectrum (**A**) of S-rGO/CuS, FT-IR spectrum (**B**) of CuS (a), S-rGO (b), S-rGO/CuS (c), TEM images of low (**C**) and high magnification of (**D**) S-rGO/CuS.

The oxidation state of Cu and S in the as-prepared bulk CuS was analyzed by XPS. The high resolution XPS spectra of the Cu 2p and S 2p core levels for the CuS (covellite) was displayed in Fig. 3A,B. The high resolution Cu 2p spectrum of the CuS was revealed the binding energies at 932.4 eV and 952.4 eV corresponding to the Cu 2p_3/2_ and 2p_1/2_. The Cu 2p_3/2_ spectrum of CuS (covellite) revealed a single peak at 932.4 eV for the single oxidation state of Cu (I), which are in good accordance with previously published values for Cu (I)^6^. Further, the high resolution S 2p spectra of CuS is distinguished by three typical peaks of CuS. The Gaussian fitting profile reveals that there are distinct S sites in the CuS. The signals at 161.8 and 162.7 eV attributed to the S^2−^ and S_2_^2−^, respectively^6^. This result was confirmed that the sulfur was present as two chemical environment viz., trigonal bipyramidal and disulfide bridge in CuS (Fig. 1). The Cu-S bonding characteristics were further confirmed by FT-IR analysis. Figure 4B displays the FT-IR spectrum of CuS (a), where the peak at 618.3 cm^−1^ is corresponding to the stretching vibrations of Cu-S^29^. From the XRD patterns, we clearly confirmed the formation of CuS, which exhibited that the CuS was crystallized in hexagonal close packing (hcp) system with the space group of P63/mmc (Fig. S4). The observed peaks are well matched with the reference pattern (JCPDS no. 79–2321) and there is no impurity peaks were observed^30^. These results are confirmed that the CuS was successfully synthesized by sonochemical process and the structural characteristics are strongly resemblance to the reported results of CuS (covellite). Afterwards, the CuS was synthesized accompanied with S-rGO by sonochemical process. Interestingly, the S-rGO was considerably changing the structural behavior of CuS and that was highly electrochemically active towards the glucose oxidation.

In order to confirm the structural transformation of the as-prepared S-rGO/CuS, the sequence of physical characterization was examined. At first, the XRD analysis was evaluated to investigate the structural behavior of the S-rGO/CuS. Unfortunately, the XRD patterns didn’t show any considerable changes from the bulk CuS (covellite). The obtained diffraction peaks (2θ) at 27.7, 29.3, 31.8, 32.8, 48.0, 52.7 and 59.3 are attributed to the (101) (102) (103) (006) (110) (108) and (116) where the additional peak appeared at 26.5 (2θ) corresponds to the S-rGO. Hence, the as-prepared S-rGO/CuS was clearly matched with the bulk CuS (covellite) (Fig. S5). Then, the Raman spectra and FT-IR spectra were taken into account for the structural transformation. Figure 4A shows the Raman spectra of S-rGO/CuS, which shows the typical D and G band characteristics for the S-rGO and shows the peak at 251.2 and 465.4 cm^−1^ for the vibrations of Cu-S and S-S (disulfide) respectively^29, 31^. Figure 4B displays the FT-IR spectra of S-rGO/CuS (c), which also revealed that the peaks for the S-rGO and CuS (covellite). Accordingly, these characterizations are evinced that the as-prepared S-rGO/CuS was clearly matched with the S-rGO and bulk CuS. However, the XPS spectra showed that there was some structural transformation from CuS to Cu_x_S. The valance of Cu and S was considerably changed for the S-rGO/CuS from CuS (covellite)^30, 32^. Figure 3C,D shows the high resolution XPS spectra of S-rGO/CuS, where the two peaks centered at 932.8 and 952.9 eV for the Cu 2p_3/2_ and 2p_1/2_. The Cu 2p_3/2_ was further deconvoluted into two peaks, the signals at 932.8 and 934.5 eV are attributed to the Cu (I) and Cu (II) sites^30, 33^. The peak areas of the two signals are in the ratio of 2:1 for the Cu (I) and Cu (II), respectively. On the other hand, the high resolution S 2p spectrum shows the peak at 161.5 and 162.4 eV for S^2−^ and S_2_^2−^, respectively. The negative shifts of binding energy suggests that the S-rGO/CuS is somewhat related to the digenite^33^. The S 2p peak at 168.5 eV was attributed to the adventitious surface oxidation of the S-rGO/CuS whereas the CuS (covellite) shows at 168.8 eV. This XPS results suggested that the structure of as-prepared S-rGO/CuS was changed some extent to the digenite. However, other characterizations didn’t exhibit any considerable responses. Hence, the surface of the S-rGO/CuS was oxidized to digenite phase. These studies resulted that the S-rGO has the ability to act as a mild oxidizing agent to oxidize the CuS. This result was in accordance with our previous report where sulfur doped carbon was act as an oxidizing agent while synthesize the nickel hydroxide^12^. Furthermore, the surface morphology of S-rGO/CuS was probed by HR-TEM and shown in Fig. 4C,D. The TEM images of S-rGO/CuS revealed as an aggregated spherical shape of CuS distributed on S-rGO surface. Wherein, the S-rGO sheets are clustered as a bundle of flake like shape and helps to the uniform distribution of CuS. Further, the lattice fringes of S-rGO/CuS exhibits more information about crystallinity. The high magnification of S-rGO/CuS reveals various planes which correspond to the S-rGO and CuS. Depending on the stoichiometric ratio, the electrochemical behavior of CuS was varied. This as-prepared bulk CuS (covellite) and S-rGO/CuS was further investigated for the electrochemical characterizations.

### Electrochemical properties of S-rGO/CuS for glucose oxidation

**Figure 5:**
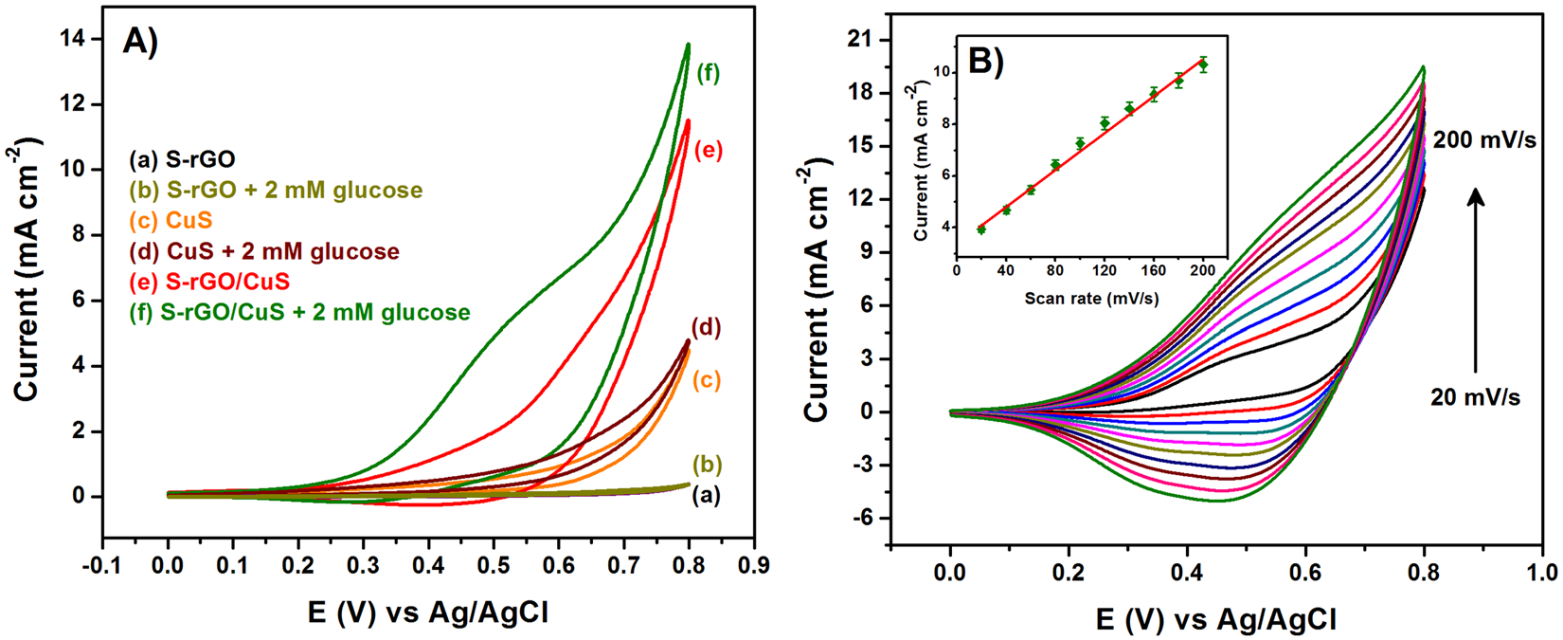
CV curves of glucose oxidation at S-rGO (a,b), CuS (c,d), S-rGO/CuS (e,f) in the absence and presence of 2 mM glucose in 0.1 M NaOH; scan rate 50 mV s^−1^ (**A**). CV responses of the glucose oxidation at S-rGO/CuS in 0.1 M NaOH at various scan rates ranging from 20–200 mVs^−1^, inset shows the corresponding plot for the oxidation peak current vs. scan rate (**B**).

The electrochemical properties of the S-rGO/CuS modified GCE and bulk CuS/GCE was examined by cyclic voltammetry (CV) in 0.1 M NaOH at a scan rate of 50 mV s^−1^. Figure 5A shows the CVs of bulk CuS/GCE (c) and S-rGO/CuS/GCE (e) which reveals an oxidation of CuS that started at +0.3 V corresponds to the formation of Cu (II) on the surface of both bulk CuS and S-rGO/CuS. This oxidized product was further reduced at +0.38 V and retained its original surface structure. However, the redox peak current of S-rGO/CuS/GCE was higher than that of bulk CuS/GCE. This high oxidation peak current of S-rGO/CuS/GCE was experienced from the local structural transformation of CuS. As stated earlier, the valance state of Cu in S-rGO/CuS was differing from the bulk CuS. Hence, the high valance of Cu(II) in S-rGO/CuS was reason for the high electrochemical activity. In contrast, there is no obvious effect was observed for the S-rGO (a), it exhibited only low background current from the double layer capacitance.

Further, these modified electrodes are explored to assess the glucose oxidation in 0.1 M NaOH containing 2 mM glucose. Figure 5A shows the CV responses of S-rGO/GCE (b), bulk CuS/GCE (d) and S-rGO/CuS/GCE (f) in the presence of 2 mM glucose. Wherein, the S-rGO/CuS/GCE (f) revealed a high electrocatalytic response towards the glucose oxidation when compare to other modified electrodes. The glucose oxidation at S-rGO/CuS/GCE (f) achieves the very low potential of 0.32 V (onset potential) accompanied with the Cu(I)/Cu(II) redox couple. The typical oxidation of glucose at the S-rGO/CuS/GCE is electrocatalyzed by the Cu(I)/Cu(II) redox couple, according to the following reactions^34^:

1 $${\rm{Cu}}\,({\rm{I}})\to {\rm{Cu}}\,({\rm{II}})+{{\rm{e}}}^{-}$$

2 $${\rm{Cu}}\,({\rm{II}})+{\rm{glucose}}\to {\rm{Cu}}\,({\rm{I}})+{\rm{gluconolactone}}$$

### Effect of scan rate

The effect of scan rate was investigated to find out the reaction kinetics of S-rGO/CuS/GCE in 0.1 M NaOH electrolyte containing 2 mM glucose. Figure 5B displays the CV responses of glucose oxidation at S-rGO/CuS/GCE for different scan rates ranging from 20 to 200 mVs^−1^. The oxidation (anodic) and reduction (cathodic) peak current of glucose at S-rGO/CuS/GCE was increased with increasing the scan rate. Herein, the reduction peak current was fully associated with the reduction of Cu (II) to Cu (I) because of the irreversible reaction of glucose. Hence, the anodic peak current was plotted against the scan rates and shown in Fig. 5B (inset). This anodic peak current was linear over the scan rates with the correlation coefficient of 0.9906. From the obtained value, we have concluded that the glucose oxidation at S-rGO/CuS/GCE was adsorption controlled process.

### Amperometric determination of glucose

**Figure 6:**
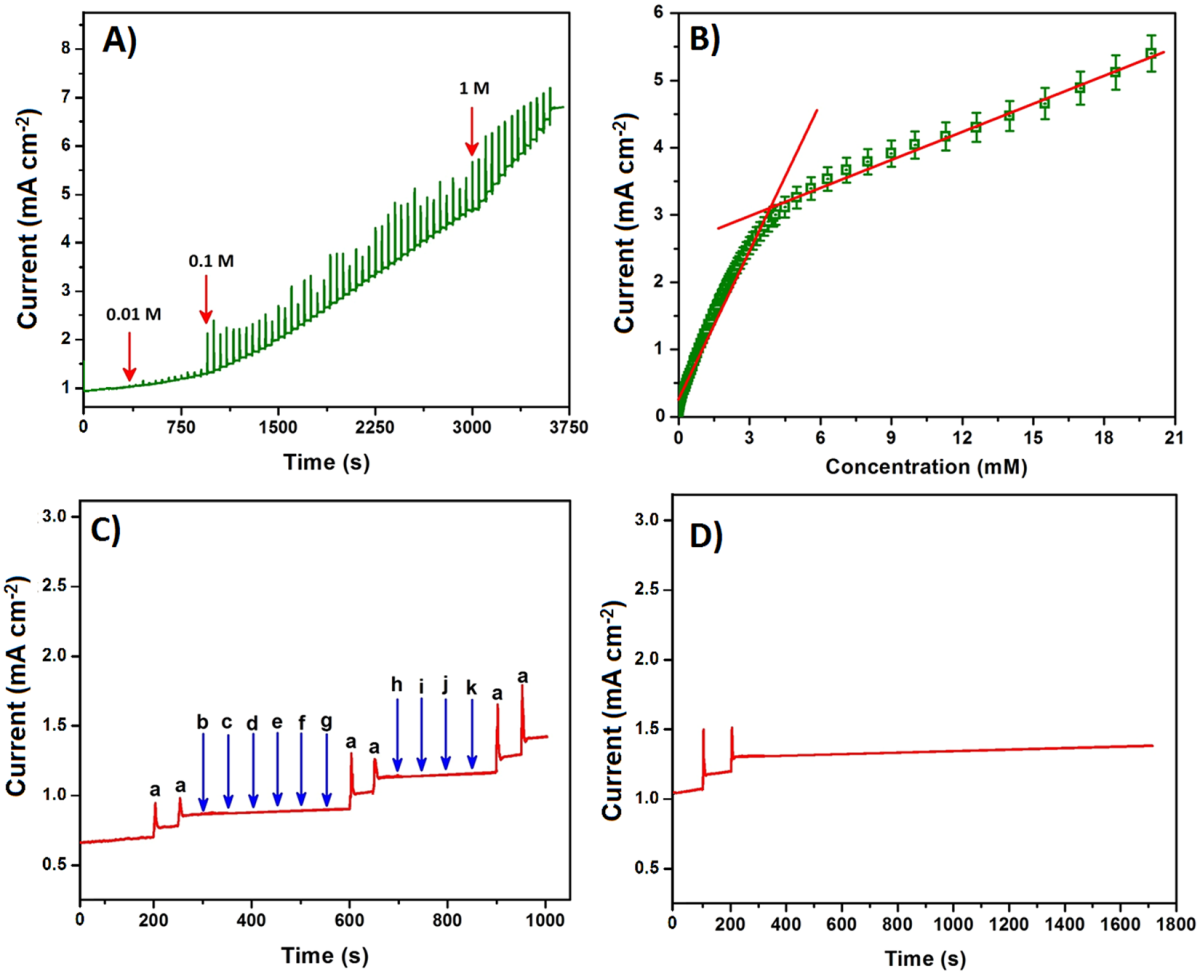
Amperometric responses of the glucose oxidation at S-rGO/CuS/RDE with the successive additions of various concentration of the glucose from 100 nM to 21.3 mM in 0.1 M NaOH at an applied potential of 0.48 V (**A**). The corresponding calibration plot of oxidation peak current vs. concentration of glucose (**B**). Amperometric signal for the glucose (a) oxidation at S-rGO/CuS/RDE with the interferences such as sucrose (b), fructose (c), lactose (d), galactose (e), Na^+^ (f), NO_3_^−^ (g), H_2_O_2_ (h), UA (i), DA (j) and AA (k) in 0.1 M NaOH (**C**). The amperometric profile for the stability test carried out at S-rGO/CuS/RDE with the addition of 0.2 mM glucose in 0.1 M NaOH up to 1400 s (**D**).

The electrocatalytic determination of the glucose was investigated on S-rGO/CuS/RDE by the amperometry technique (Fig. 6A). The amperometric study was carried out in 0.1 M NaOH with the convection mode (1500 rpm) and the successive additions of glucose in the range from 0.0001 to 21.3 mM at 0.48 V. The S-rGO/CuS/RDE revealed the sharp response signal for the addition of 100 nM glucose within 0.8 s, which is the fastest response for the glucose oxidation at 1500 rpm. Afterwards, the electrocatalytic responses were attained the steady-state current. The electrocatalytic oxidation current of glucose was increased when increased the concentration. These oxidation current responses were plotted against the concentration of glucose. Figure 6B displays the calibration plot of glucose oxidation which exhibits the two linear concentration ranges from 0.0001 to 3.88 mM and 3.88 mM to 20.17 mM. These two linear responses were observed from the adsorption controlled kinetics of glucose oxidation at S-rGO/CuS/RDE. In which, the low concentration range facilitates that the oxidized product was quickly moved away from the electrode surface. Hence, the oxidation peak current was higher for the low concentrations which lead to the first linear limit with the correlation coefficient of 0.9866. On the other hand, the oxidation peak current was decreased for the high concentrations, because the oxidized product does not escape easily from the electrode surface. Moreover, the high concentration of glucose was limiting the diffusion of analyte. Hence, it creates an internal resistance at electrode surface. Therefore, the oxidation current was lower when compared with first linear range. From these observed parameters, the sensitivity of glucose oxidation at S-rGO/CuS/RDE was calculated as 429.4 µAmM^−1^ cm^−2^ and 76.27 µAmM^−1^ cm^−2^ for the first and second linear ranges, respectively. From this first linear range the limit of detection (LOD) was calculated as 32 nM. The wide linear range (~20 mM) and acceptable sensitivity was certified that the proposed sensor material was highly suitable for the real sample analysis, because the observed values are in accordance with the human blood glucose concentrations (5–8 mM)^35^.

The analytical performances of the developed non-enzymatic S-rGO/CuS glucose sensor electrode material were compared with other non-enzymatic glucose sensor electrode materials (Table 1). The developed sensor material was compared with the analytical parameters such as potential, sensitivity, linear range and LOD of the other reported materials. Only seldom glucose sensors were reported based on the copper sulfide matrix. However, S-rGO/CuS was exhibited acceptable performance when compare with other reports. Moreover, the stability is one of the main factor while developing the sensor electrodes; so, the amperometric study was investigated to examine the operational stability of S-rGO/CuS/RDE. Figure 6D shows the amperometric response of S-rGO/CuS/RDE which revealed sharp responses for the additions of 0.2 M glucose in 0.1 M NaOH. Afterwards, the steady state current was achieved for 1400 s which showed that the S-rGO/CuS glucose sensor electrode has good operational stability.

**Table 1:** Comparison of the analytical performance of the S-rGO/CuS sensor electrode with previously reported non-enzymatic glucose sensor electrodes.

Electrode	Potential (V)	Sensitivity (µA mM^−1^ cm^−2^)	Linear range (up to, mM)	Detection limit (µM)	ref
Cu-CuO nanowire	0.3	—	12	500	37
CuO nanorods-graphite	0.60	371.4	8.0	4.0	38
CuO nanospheres	0.60	404.5	2.6	1.0	39
Cu_2_S nanocrystal-DWCNT/GCE	0.5	35	11.7	1.0	40
CuO nanocubes/graphene	0.59	1360	4	0.7	41
CuO nanoplatelets	0.55	3490.7	0.8	0.5	42
Copper nanocluster- carbon nanotubes	0.65	251.4	3.5	0.21	43
Cu_2_O nanospindle/MWCNTs	0.4	2143	2.5	0.2	44
CuO nanowires	0.33	0.49	2.0	0.049	45
CuS	0.45	3135	2.5	0.045	13
Sphere-like CuS microcrystals	—	117.3	12	0.015	46
CuS nanotubes	—	11100	0.005	—	47
S-rGO/CuS	0.48	429.4	20.17	0.032	This work

### Interference studies

The analytical parameters of non-enzymatic S-rGO/CuS sensor electrode were evaluated in the aspect of stability and sensitivity. However, the selectivity also plays an important role in the development of non-enzymatic glucose sensor. Hence, the glucose was determined in the presence of other co-interfering substances. In general, the glucose was co-existing with dopamine (DA), uric acid (UA), ascorbic acid (AA) and some other carbohydrates in blood^36^. Moreover, the hydrogen peroxide (H_2_O_2_) was highly interfere with glucose oxidation; because, H_2_O_2_ was electrochemically oxidized at closer to glucose oxidation potential. Hence, the glucose (a) oxidation was examined in the presence of sucrose (b), fructose (c), lactose (d), galactose (e), Na^+^ (f), NO_3_^−^ (g), H_2_O_2_ (h), UA (i), DA (j) and AA (k) in 0.1 M NaOH. The interfering substances are taken 30 times lower than that of glucose concentrations, because the physiological level of glucose concentration is 3–8 mM^12^. Figure 6C displays the amperometric responses of glucose oxidation in the presence of interfering substances in 0.1 M NaOH at 0.48 V. As shown in Fig. 6C, the sharp responses were observed for the additions of 0.05 mM glucose (a) and no obvious responses were observed for the additions of 0.002 mM of other interferents. This study exhibited that the S-rGO/CuS/RDE revealed high selectively in the presence of co-interfering species.

### Real sample analysis

To investigate the commercial viability of the developed non-enzymatic S-rGO/CuS glucose sensor, the real sample analysis was performed. Here, the determination of glucose concentrations was carried out in biological samples such as human blood serum, urine and saliva. All these biological samples were collected from normal patients. As per the literature, the approximate glucose level in these samples are 4.95 mM (blood serum), 0.8 mM (urine) and 0.7 mM for non-diabetic patients^48–51^. Further, these samples were diluted to three times by DI water and directly applied for the detection of glucose in 0.1 M NaOH electrolyte solution. The amperometric technique was exploited to investigate the concentrations of glucose in saliva, urine, blood serum samples by different time interval. The experimental procedures are followed here as same as in section of amperometric determination of glucose. The obtained recoveries of glucose concentration in biological samples were given in Table 2.

**Table 2:** The recoveries for the determination of glucose in human saliva, urine and blood serum.

Real sample	Add (µM)	Found^a^ (µM)	Recovery (%)	RSD^b^ (%)
Blood serum	—	11.2	—	—
	10	21.1	99.5	3.1
Saliva	—	0	—	—
	10	9.6	96.0	3.2
Urine	—	0.4	—	—
	10	10.3	99.03	2.81

^a^Standard deviation method and ^b^Relative standard deviation with three repetitive measurements.

The developed S-rGO/CuS glucose sensor electrode has attained the acceptable recovery ranging from 95 to 99% for the biological samples. Therefore, the developed S-rGO/CuS glucose sensor electrode has to be applied for the real time sensing of glucose in real samples analysis. Moreover, we have validated our proposed material with the hospital samples and compared the values with relative error. Five different hospital samples were taken from the normal person and patient. These samples were analyzed by the proposed material and the results are compared with the hospital results that show the relative error percentage is 3–7% for the hospital samples. This error is maximum for the calibrated glucose sensor device, however, the <5% error is acceptable for the initial lab analysis.

## Conclusions

We demonstrated the sonochemical route to synthesize the S-rGO and CuS nanocomposite. Among the reported chemical routes, the sonochemical method provides highly efficient and rapid way to produce nanomaterials. Moreover, it is an alternative technique to prepare the sulfur doped graphene by simplest way. The mild oxidizing power of S-rGO was described towards the surface oxidation of CuS. The electrocatalytic activity of CuS was systematically addressed by the change in crystal phase. The S-rGO/CuS composite was fabricated as glucose sensor electrode and determined the glucose by a wide linear concentration range of 0.0001–3.88 mM and 3.88–20.17 mM, and a low-level detection limit of 32 nM. Furthermore, it exhibited a good stability and also excellent selectivity in the potentially co-interfering ions and biological substances. Moreover, the practical applicability of the S-rGO/CuS glucose sensor electrode was confirmed towards the determination of glucose in biological samples such as human saliva, urine and blood serum. In addition, the proposed material was validated by hospital samples that show the relative error percentage is 3–7%. This error is maximum for the calibrated glucose sensor device, however, the <5% error is acceptable for the initial lab analysis.

## Experimental section

### Materials

Graphite powder (for the preparation of GO), copper chloride dihydrate (CuCl_2_.2H_2_O), sodium sulfide (Na_2_S), hydrogen peroxide (H_2_O_2_), fructose, sucrose, galactose, lactose, sodium nitrate, uric acid, ascorbic acid, dopamine, NaOH and all other chemicals were purchased from Sigma-Aldrich, Alfa Aesar, Fluka and Wako chemicals and used as received without any purification.

### Apparatus and Electrochemical measurements

The structural characterizations of CuS and S-rGO/CuS was analyzed by X-ray diffraction pattern analysis (XRD), XPERT-PRO (PANalytical B.V., The Netherlands) with CuKα radiation (λ = 1.5406 Å), Fourier transform infrared spectroscopy (FT-IR–JASCO FT/IR-6600), Raman spectra (NT-MDT, NTEGRA SPECTRA) and the surface morphology was probed by using high-resolution transmission electron microscopy (HR-TEM- TECNAI G^2^). All the electrochemical studies were carried out in CHI 405 A electrochemical work station (CH Instruments, USA) and the rotating disk electrode (RDE) AFMSRX (PINE instruments, USA) or glassy carbon electrode (GCE) as a working electrode, platinum wire as a counter electrode and saturated Ag/AgCl (saturated KCl) as a reference electrode.

### Preparation of S-rGO/CuS

The GO was prepared by modified Hummers method from the graphite powder. The 3 mg of as-prepared GO was re-dispersed in 5 mL of de-ionized water. This GO suspension was sonicated for 30 min then the 1 mL of 0.5 M Na_2_S was added to the GO suspension. This mixture was sonicated for an hour at 100 °C. Now the GO solution turned to S doped reduced graphene oxide flakes. Afterwards, the S-rGO suspension was allowed to cool at room temperature and the S-rGO was separated by centrifugation. Then, 1 mg of S-rGO flakes was re-dispersed 1 mL of de-ionized water and sonicated with the addition of 1 mL of 5 mM CuCl_2_ solution for 2 hours. Now, the copper ions are evenly distributed on the surface of S-rGO. Finally, 1 mL of 0.5 M Na_2_S was added to this solution and sonicated for an hour. Then the precipitate was centrifuged and washed with copious amount of water to remove residual impurities. This S-rGO/CuS was used to further physicochemical characterizations. For comparison, the S-rGO and bulk CuS was prepared by the same method. The overall synthesis procedure was given in Fig. 2.

### Fabrication of modified electrodes

The as-prepared materials (S-rGO, bulk CuS and S-rGO/CuS) are re-dispersed in de-ionized water and sonicated for 30 min. Then, about 8 µL of those suspensions was drop coated on the surface of glassy carbon electrode (GCE- working area = 0.07 cm^2^) or 15 µL on RDE (RDE-working area 0.196 cm^2^). This drop coated electrodes are allowed to dry in room temperature and then gently rinsed by de-ionized water to remove the loosely bounded particles. These modified electrodes were further used for the electrochemical characterizations.

### Preparation of real samples for glucose detection

The as-fabricated S-rGO/CuS electrode was utilized for glucose detection in biological samples such as human blood serum, urine and saliva. These samples were collected from the healthy patients and diluted 3 times with DI water. The prepared real samples were added into 0.1 M NaOH with different time interval. Amperometry technique was used to monitor the glucose oxidation on S-rGO/CuS/RDE for real samples by applying constant potential of 0.48 V with the rotation speed of 1500 rpm.

## Electronic supplementary material

Supplementary Information 
