# Supplementary material for: Sonochemical Synthesis of Sulfur Doped Reduced Graphene Oxide Supported CuS Nanoparticles for the Non-Enzymatic Glucose Sensor Applications
Source: Sci Rep. 2017 May 30;7:2494. doi: 10.1038/s41598-017-02479-5 (PMC5449390; doi:10.1038/s41598-017-02479-5)
Supplement: Supplementary file 1 — Supplementary Information [file 41598_2017_2479_MOESM1_ESM.pdf]

Supporting information

Sonochemical synthesis of sulfur doped reduced graphene oxide supported CuS nanoparticles for the non-enzymatic glucose sensor applications

Natarajan Karikalan[a], Raj Karthik[a], Shen-Ming Chen*[a], Chelladurai Karuppiah[a,b] and Arumugam Elangovan[c]

[a]Electroanalysis and Bioelectrochemistry Lab, Department of Chemical Engineering and Biotechnology, National Taipei University of Technology, No.1, Section 3, Chung-Hsiao East Road, Taipei 106, Taiwan (R.O.C).

[b]Department of Chemistry, National Taiwan University, No. 1, Section 4, Roosevelt Road, Taipei 106, Taiwan, ROC.

[c]Department of Chemistry, Thiagarajar college, Madurai, Tamilnadu 625009, India.

*Corresponding author. Tel: +886 2270 17147, fax: +886 2270 25238.

E-mail address: smchen78@ms15.hinet.net (SM Chen)

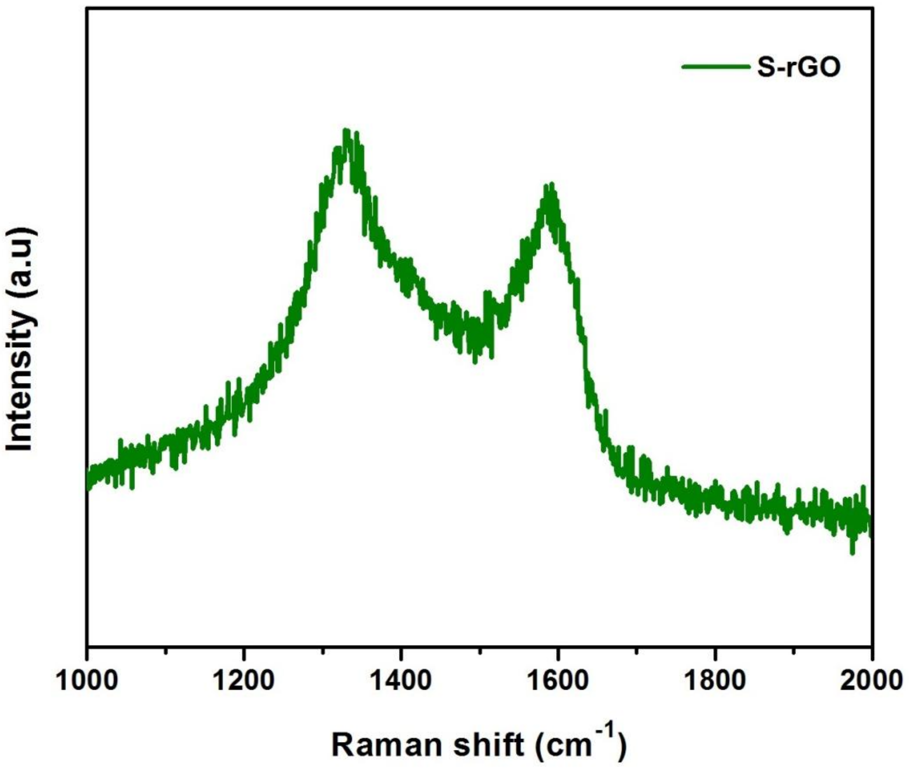

Fig S1. Raman spectrum of S-rGO

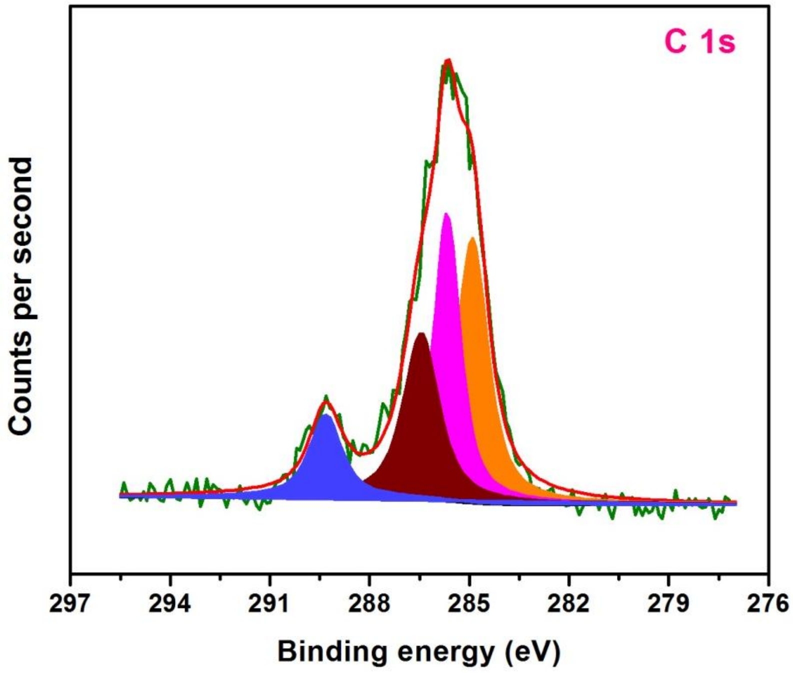

Fig S2. High resolution C 1s XPS spectrum of S-rGO

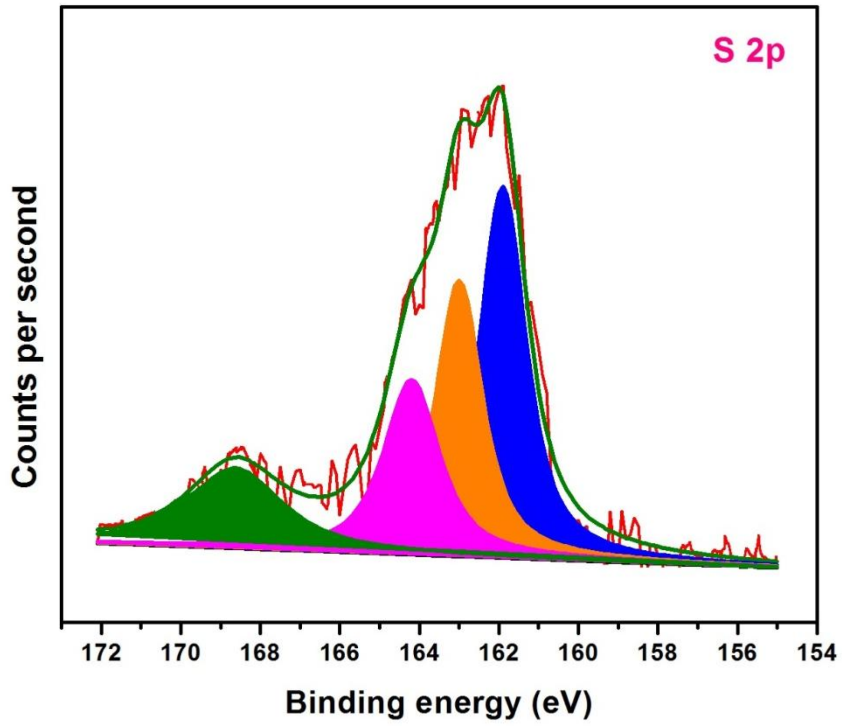

Fig S3. High resolution S 2p XPS spectrum of S-rGO

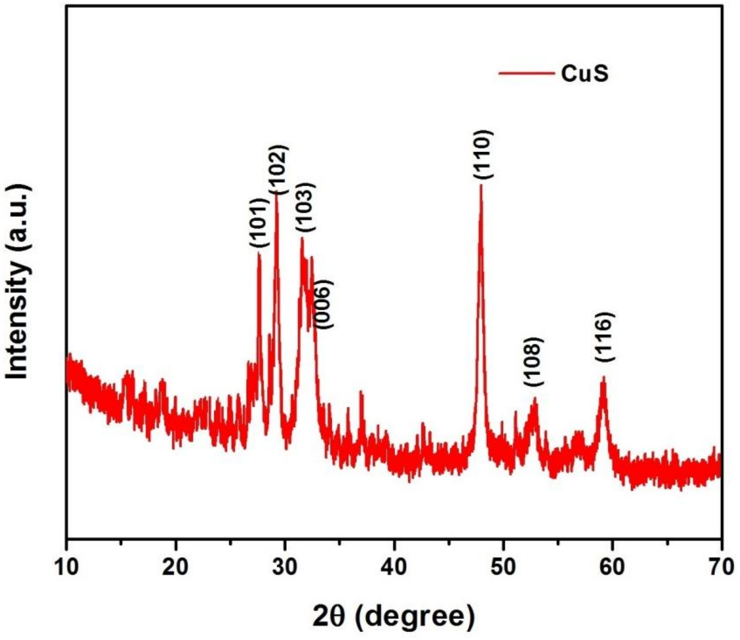

Fig S4. XRD patterns of bulk CuS

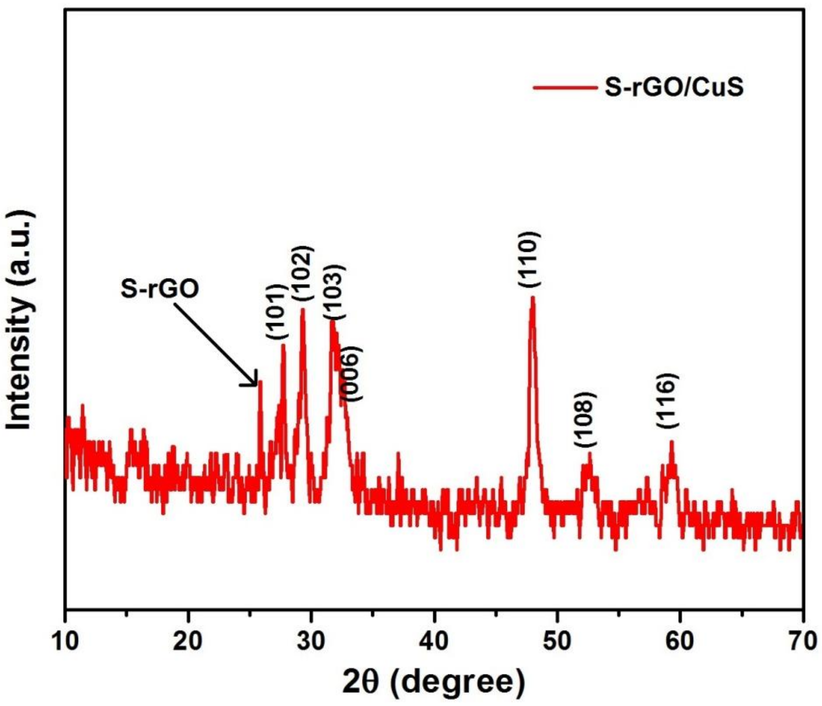

Fig S5. XRD patterns of S-rGO/CuS
